# Demographic and Radiographic Characteristics Associated with the Occurrence of Impacted Third Molars in Indonesian Patients: A Retrospective Study

**DOI:** 10.3390/dj12070210

**Published:** 2024-07-09

**Authors:** Indra Hadikrishna, Melita Sylvyana, Madhuri Pattamatta, Folli Mulyawati, Tantry Maulina

**Affiliations:** 1 Oral and Maxillofacial Surgery Department, Faculty of Dentistry, Universitas Padjadjaran, Bandung 40132, Indonesia; indra.hadikrishna@unpad.ac.id; 2 Oral and Maxillofacial Surgery Department, Hasan Sadikin Hospital, Bandung 40161, Indonesia; melita.sylvyana@gmail.com; 3 Dentistry Department, Faculty of Medical Sciences, Radboud University Medical Center, 6525 EX Nijmegen, The Netherlands; madhuri.pattamatta@radboudumc.nl; 4 Dental Department, Kota Serang Regional Public Hospital, Serang 42124, Indonesia; folli.mulyawati@bantenprov.go.id

**Keywords:** third molar, impacted tooth, third molar impaction, radiographic, demographic factors

## Abstract

An impacted third molar is one of the most common abnormalities of the tooth position, impacting patients and their quality of life. Based on the impact and the invasive removal procedure, this study aimed to evaluate the characteristics of impacted third molars based on their radiographic features as well as their association with demographic characteristics. Outpatient dental records of the oral and maxillofacial surgery department of Hasan Sadikin Hospital, Bandung, Indonesia, from 1 January 2018 to 31 December 2019, were sorted, and relevant clinical and demographic data and panoramic radiographic examination results were extracted from these records. All data were then tabulated and analyzed by using SPSS version 29. As many as 3019 impacted third molars were identified. Our findings suggested the association between age to the occurrence of impacted third molars where patients aged between 17 and 29 years old showed high occurrences of impacted third molars. Male patients with impacted third molars are more likely to have multiple impacted third molars, while this risk decreases in females. Radiographic examination showed that the mesioangular position is the most common position for mandibular third molars. The variation in high occurrences of the impacted third molar is associated with several demographic factors.

## 1. Introduction

Impacted third molars, one of the most common problems in the oral cavity, can be caused by several factors, with the lack of space in the dental arch being one of the most common causes [1]. The global prevalence of impacted third molars varies from 16% to 68.6%, even though a slightly higher prevalence (73%) was found in the young European population [2]. As defined by Andreasen et al., the term “impacted” can be defined as “*a cessation of the eruption of a tooth caused by a clinically or radiographically detectable physical impediment in the eruption path or by an ectopic position of the tooth*” [3]. Impacted third molars have been known for their medical consequences within the orofacial area, namely, pain, swelling, caries formation on the adjacent tooth, periodontal disease, as well as the formation of dentigerous cysts [4,5]. Due to its various clinical impacts, the quality of life of impacted third molar(s) patients is often negatively influenced [5,6].

Due to the negative impact of impacted third molars, removal of impacted third molar has become one of the most performed oral surgery procedures [7]. Studies are continuously performed to investigate the occurrence of impacted third molars and its association with clinical, as well as sociodemographic, factors [8,9,10,11,12] to gain a better understanding as well as to prevent possible complications during removal procedures [13]. In a study conducted by Adeola et al. (2023), the occurrence of impacted third molar was associated with age where higher incidence was observed in the 21–29-year-old age group [11]. In a study performed by Al-Gunaid (2020), it was revealed that sex is associated with the behavior of impacted third molars. For example, women are most likely to have more upright posterior teeth and less inclined third molars [12]. In another study conducted by Ahmad et al. (2021) regarding the pattern of third molars impaction, it was found that impacted third molars were more likely to occur in women [13].

Additionally, studies regarding the radiographic characteristics of impacted third molars are continuously conducted as well [14,15,16,17]. Radiographic examination of impacted third molars can be used to predict possible complications, and as a decision-making tool, especially when the impacted third molar is asymptomatic [15,16]. In a study conducted by Fernandes et al. (2022), it was stated that there are three radiographic signs that can be used to determine possible alterations of the hard tissue and the necessity of third molar removal. Those signs are (1) marginal bone loss in the distal area of the adjacent second molar; (2) increased radiolucent area that surrounds the crown of the impacted third molar; and (3) bone resorption in the adjacent second molar [16]. This indicates the importance of including radiographic examination in the investigation of impacted third molars and their removal procedure.

Interestingly, regardless of the continuing studies, the characteristics of the impacted third molar and its association with the possible risk factors still show some variations. For example, even though previous studies showed an association between sex and the occurrence of impacted third molar [12,18], a study by Al-Dajani et al. (2017) showed the opposite, where no significant difference between males and females regarding impaction frequency, depth level, and angulation [19]. A similar result was also shown by Zaman et al. (2021) in their study regarding the pattern of third molar impaction where no significant gender predominance was found [20]. Based on the inconclusive results of the previous studies, this study aimed to evaluate the characteristics of impacted third molars based on their radiographic features as well as their association with demographic characteristics.

## 2. Material and Methods

This retrospective study recruited dental records of patients from the outpatient clinic of the Oral and Maxillofacial Surgery of Hasan Sadikin Dental Hospital who came to have their third molar removal procedure from 1 January 2018 to 31 December 2019. Due to the nature of the study, no ethical approval was required. Prior to removal procedure of the impacted third molars, all patients signed an informed consent, consenting for their approval regarding the usage of their data for research purposes and any related future publications. Informed consent for patients under 18 years of age was also signed by their parent or legal guardian. Data regarding age, sex, number of impacted teeth, types of impacted teeth, and radiographic examination results were extracted from the dental record. Considering the most common age for the eruption of the third molars, only the data of those aged 17 years and above were included in this study. Therefore, the inclusion criteria of the current study were dental records of patients who came during this study period aged 17 years old or older and gave their consent for the usage of their data to be included in any future study related to their condition and treatment. If the dental record did not contain any of the required data, it was excluded from the study. 

### 2.1. Radiographic Examination Identification

All patients had to go through a panoramic radiographic examination. The position of the impacted third molars was assessed based on the Pell and Gregory classification and the Pederson Index (the visualization of the Pederson index has been thoroughly described in Bali et al. [21]). The Pederson index offered a modification to the classification provided by Pell and Gregory. It may not correlate with the actual level of difficulty when used on an easy-to-moderate level of difficulty as the judgment is mainly based on local anatomy and radiographs [22]. The radiographic examination results were used to investigate the characteristics of the impacted third molar from a radiographic point of view. 

### 2.2. Maxillary Third Molar

For relative depth, the radiographic examination findings on the maxillary third molar were classified based on the highest position of the third molar. If the highest position of the third molar was at the same level or above the occlusal plane of the second molar, then it was classified as class A, if it was between the occlusal plane and cervical line of the second molar, then it was classified as class B, and if it is below the cervical line of the second molar, then it was considered as class C. For maxillary impacted third molar, sinus approximation identification was conducted. If there was no bone or if there was a thin bone between the impacted third molar and the maxillary sinus, then the condition was identified as “sinus approximation”. If there was a bone that is at least 2 mm in thickness between the impacted third molar and the maxillary sinus, then the condition was identified as “no sinus approximation” [3]. If the sinus approximation identification cannot be concluded, then the impacted third molar will be identified as “undefined”. 

### 2.3. Mandibular Third Molar

For the mandibular impacted third molars, the spatial relationship (the position of the impacted molar) was classified into mesioangular, horizontal, vertical, distoangular, and inverted. This was based on the Winter classification as described in the study by Kim et al. (2019) [23]. Similar to the upper third molar, a relative depth identification based on the highest position of the mandibular impacted third molars to the second molar was also performed. Lastly, the mandibular impacted third molars were also classified based on the space available between the ramus and the distal margin of the second molar (ramus relationship), as described in the study of Alsaegh et al. (2022) [24].

### 2.4. Statistical Analysis

All data were then analyzed by using SPSS version 29 (IBM Corp. Released 2023. IBM SPSS Statistics for Macintosh, Version 29.0.2.0 Armonk, NY, USA: IBM Corp). The analysis of frequencies in the current study was performed by using Crosstabs analysis. Significant differences were tested by Chi-square analysis, and Odds ratio calculation was performed by binary logistic regression analysis.

## 3. Results

Eight hundred and eighty-five patients’ dental records were retrieved and analyzed in this study. Out of the 885 patients who underwent the third-molar dental procedure, 596 of them were females and 633 of them were between the age of 17 and 30 years old (age range 17–69 years old; mean age is 27.7 years old; SD = 9.3). Another highlight of the findings is that they are age-associated, and that those who are aged between 17 and 30 years old (76.62%) have a higher frequency of having 4 to 6 impacted teeth compared to those who are aged above 30 years. For those who are aged above 30 years old, there was no likelihood as to whether they will have 1–3 impacted teeth or 4–6 impacted teeth. The distribution of patients based on their demographic and clinical characteristics can be viewed in Table 1.

Sex and age of the patients were also used to reveal the proportion of bilateral and unilateral impacted third molars (see Table 1). From the figure, it can be seen that bilateral impacted third molars were most commonly found in female patients, and those aged 17–30 years old age range.

Out of the 885 hundreds dental records, 3019 impacted third molars were identified. Out of the 3019 impacted third molars, 48.29% (1458) were in the maxilla, and 51.71% (1561) were in the mandible. The impacted third molars were also classified based on the findings of the radiographical examination. Based on the findings, it was found that for the maxillary third molar, most impacted teeth had sinus approximation (right upper third molar: 468 teeth; left upper third molar 510 teeth). Additionally, when it comes to relative depth examination for the right upper third molar, most teeth were classified as class A, while the left upper third molars were mostly classified as class C. As for lower third molars, most impacted third molars on both sides were classified into class A, and class II for the ramus relationship classification (Table 2).

From Table 2, it can also be observed that the most current position for an impacted mandibular third molar is the mesioangular position followed by the horizontal position. It is interesting to note that when maxillary impacted third molars are compared with mandibular third molars, maxillary impacted third molars were more likely to reside below the cervical line of the second molar and therefore classified to Class C, while the mandibular third molars were more likely to reside in the same level as the occlusal plane of the second molars and therefore classified into class A.

Additionally, an analysis of the association of the demographical factors to the occurrence of the impacted third molars was also performed. There is a significant difference (*p* < 0.001) regarding the occurrence of impacted third molar when associated with age, of which impacted third molars are more likely to occur in those aged 17–30 years old. Those who are 17–30 years old were 1.05 times (CI 95%; 0.88–1.21) more likely to have additional impacted teeth while those who are older than 30 years old have decreased odds ratio (OR: 0.92; CI 95%: 0.65–1.32). In regard to sex, male patients who had impacted third molars were 1.59 times (CI 95%: 0.92–2.11) more likely to have another or additional impacted teeth, while being female decreased the odds ratio up to 0.88 times (CI 95%; 0.76–1.00).

## 4. Discussion

In general, the association between sex and age on the occurrence of impacted third molars found in the current study is in line with previous studies [11,13,25,26]. The different points of eruption between males and females might contribute to these associations. In research conducted by Rozzi (2016), it was revealed that tooth eruption occurs earlier in females than in males, especially for third molars [27]. Furthermore, in a study conducted by Milos et al. (2021), it was hypothesized that compared to the maxilla, the mandible grows more during the adolescent period and that the phenomenon occurs more in males compared to females. This hypothesis was then confirmed with the findings of their study. They also stated that craniofacial growth in males continues after the age of 18 years old [28]. These differences, of course, have the potential to cause age and sexual differences on impacted third molar occurrences considering that the impacted condition is affected by the availability of space, both in the maxilla and in the mandible. 

The current retrospective study was conducted based on the dental records of 885 patients, where more female patients (67.34%; male: female ratio = 1:2.06) were found. This result supports a previous study conducted by Ahmad et al. (2021) and Gebeyehu and Abaynew (2024) where impacted third molars were more common to be found in female than in male patients [13,29]. This is interesting as previous studies [25,30] also showed the predominance of the male sex compared to female. Additionally, it is also interesting to note that in a study conducted by Alfadil and Almajed (2020) in Saudi Arabia on 4000 patients’ orthopantomogram (OPG) radiographs of which 1014 OPGs showed the occurrence of impacted third molars, no gender predominance was identified [9]. In regard to the female predominance, it was hypothesized that this might be influenced by jaw size, hormonal changes, as well as genetic factors [29]. The eruption of the third molar is at a point when the growth of most female jaws reaches completion. On a slightly different note, male jaw growth continues during the eruption of the third molar, potentially providing more adequate room for the third molar to erupt [9].

In regard to age, in this study, the age range was divided between 17 and 30 years old and above 30 years old, with impacted third molars occurring most in the first age range. This particular result is rather similar to the findings of the study conducted by Adeola et al. (2023). In the study of 469 Nigerian patients’ dental records, Adeola et al. (2023) found that impacted third molars are most likely to occur in patients aged between 20 and 29 years old. This predominance may result from the fact that late adolescence and early adulthood are the times when impacted third molars are most likely to emerge [11]. Similarly, Shaari et al. (2023) studied the prevalence of impacted third molars in an Iraqi population and also found that impacted third molars are most likely to occur within the age range of 21 to 30 years old [26].

In the current study, no predominance regarding the maxilla and the mandible was found. This result is different from a previous study conducted by Alfadil and Almajed (2020) where impacted third molars were more commonly found in the mandible compared to the maxilla [9]. In another study conducted by Genç, Orhan, and Hincal (2022) in a North Cyprus population, impacted third molars were also found more in the mandible (70.27%) compared to the maxilla (29.73%) [25].

Clinical evaluation for the third molars’ class in the current study was based on the Pell and Gregory classification [14], with class A being the most common class found for maxillary and mandibular impacted third molars. This result is contradictory to the findings of the study conducted by Ishwarkumar et al. (2019) on a South African population. In their study, Ishwarkumar et al. (2019) found that the most common class based on the Pell and Gregory classification for the third molar impaction found in the mandible is class B (54.4%), followed by class C and class A. While for the third molars found in the maxilla, the most common class was class A, followed by class C and class B [2]. Additionally, a study conducted by Shaari et al. (2023) showed that the most common class found for mandibular impacted third molars based on Pell and Gregory classification is class II followed by class III, and class I being the least common [26]. The findings from Shaari et al.’s study (2023) are in line with the findings of the current study where class II was the most commonly found class amongst mandibular impacted third molar.

Furthermore, the radiographic evaluation on the mandibular impacted third molar in this study showed that the mesioangular position is the most common position found. This finding is in line with a previous study conducted by Adeola et al. (2023) on 469 patients in Nigerian Teaching Hospital, where the mesioangular position is the most common impaction found (36.5%) [11]. The occurrence of mesioangular-impacted third molars has been associated with a high risk of caries development, both in the second and third molars. The mesioangular position has also been associated with periodontal bone loss, distally to the adjacent second molar [31]. Therefore, the removal of mesioangular-impacted third molars in patients should be carefully performed.

In the current study, the radiographic evaluation showed that 67.08% of the impacted maxillary third molar has sinus approximation. This result is in line with a study conducted by Waseem et al. (2021). In their study, Waseem et al. (2021) reported that out of 281 maxillary impacted third molars being investigated, 66.19% showed sinus approximation [32]. Sinus approximation is recognized as one of the risk factors for post-impacted maxillary third-molar removal complications, considering the proximity of the impacted third molar to the sinus floor [33]. The proximity of the sinus floor to the apical part of the posterior teeth in the maxilla is affected by age, sinus pneumatization, and duration of the edentulous condition, aside from other factors [34]. In order to avoid such complications, identification of this condition prior to the removal of the impacted maxillary third molars is important. 

The study is not without limitations. Due to the availability of the radiographic examination result, panoramic radiographic analysis was the only radiographic analysis that we could perform. Therefore, we could only conduct our radiographic analysis on two-dimensional panoramic radiography. An analysis of the dental cone beam computed tomography (CBCT) radiographic would have provided a more thorough analysis as it provides a three-dimensional result. 

## 5. Conclusions

The current study found that there was no predominance for the occurrence of impacted third molars concerning jaw location. Sex and age predominance were found in this study where women showed a higher prevalence of impacted third molars, while those aged 17 and 30 years old also showed a higher prevalence of impacted third molars, indicating the association of the occurrence of impacted third molars to demographic factors. Radiographic evaluation on mandibular impacted third molars showed that class II is the most found class when it comes to the ramus relationship, and mesioangular position is the most common position. Lastly, class A was the most found class among all impacted third molars.

## Figures and Tables

**Table 1:** The distribution of patients based on demographic and clinical characteristics.

Category	Participant Distribution	
	**Sex**	
	**Male**	**Female**
Age		
17–30 years old	203	430
>30 years old	86	166
Number of impacted teeth		
1–3 teeth	93	181
4–6 teeth	194	415
>6 teeth	2	0
Types of impacted teeth		
Third molar only	271	539
Third molar and other teeth	15	45
Teeth other than third molar	3	12
Occurrence of third molars		
Unilateral	17	42
Bilateral	272	554
	**Age**	
	**17–30 years old**	**>30 years old**
Number of impacted teeth		
1–3 teeth	146	128
4–6 teeth	485	124
>6 teeth	2	0
Types of impacted teeth		
Third molar only	581	229
Third molar and other teeth	46	14
Teeth other than third molar	6	9
Occurrence of third molars		
Unilateral	21	38
Bilateral	612	214

**Table 2:** Distribution and clinical classification of impacted third molars based on their radiographic interpretation (Pederson classification and sinus approximation method).

**Right upper third molars (n = 722)**						
Relative depth *	Class A	Class B		Class C		Undefined
	288 (39.9%)	172 (23.8%)		255 (35.3%)		7 (1.0%)
Sinus approximation	No sinus approximation		Sinus approximation			Undefined
	246 (34.1%)		468 (64.8%)			8 (1.1%)
**Left upper third molars (n = 736)**						
Relative depth	Class A	Class B		Class C		Undefined
	290 (39.4%)	138 (18.8%)		304 (41.3%)		4 (0.5%)
Sinus approximation	No sinus approximation		Sinus approximation			Undefined
	221 (30%)		510 (69.3%)			5 (0.7%)
**Left lower third molars (n = 785)**						
Spatial relationship/Position of the impacted molar	Mesioangular	Horizontal	Vertical	Distoangular	Inverted	Undefined
	301 (38.3%)	242 (30.8%)	205 (26.1%)	8 (1.0%)	2 (0.3)	27 (3.4%)
Relative depth *	Class A	Class B		Class C		Undefined
	364 (46.4%)	282 (35.9%)		112 (14.3%)		27 (3.4%)
Ramus relationship/Available space **	Class I	Class II		Class III		Undefined
	87 (11.1%)	632 (80.5%)		39 (5.0%)		27 (3.4%)
**Right lower third molars (n = 776)**						
Spatial relationship/ Position of the impacted molar	Mesioangular	Horizontal	Vertical	Distoangular	Inverted	Undefined
	306 (39.4%)	258 (33.2%)	182 (23.5%)	13 (1.7%)	3 (0.4%)	14 (1.8%)
Relative depth	Class A	Class B		Class C		Undefined
	330 (42.5%)	286 (36.9%)		146 (18.8%)		14 (1.8%)
Ramus relationship/Available space	Class I	Class II		Class III		Undefined
	82 (10.6%)	630 (81.2%)		50 (6.4%)		14 (1.7%)

* **Relative depth:** “*Class A: the highest position of the third molar is at the same level or above the occlusal level of second mandibular molar; Class B: the highest position of the third molar is below the occlusal plane but above the cervical line of second mandibular molar; Class C: third molar is below the cervical line of second mandibular molar*;” ref. [21] Undefined: position cannot be interpreted radiographically. ** **Ramus relationship:** “*Class I: there is sufficient space between the ramus and distal margin of the second molar for the accommodation of the mesial–distal diameter of the crown of the third molar; Class II: space between the ramus and distal surface of the second molar was less than the mesial–distal diameter of the crown of the lower third molar; Class III: all or most of the third molar lay within the ramus*”; ref. [21] undefined: position cannot be interpreted radiographically.

## Data Availability

The data presented in this study are available on request from the corresponding author.
